# Early metabolite changes after melatonin treatment in neonatal rats with hypoxic-ischemic brain injury studied by *in-vivo* ^1^H MR spectroscopy

**DOI:** 10.1371/journal.pone.0185202

**Published:** 2017-09-21

**Authors:** Hester Rijkje Berger, Axel K. G. Nyman, Tora Sund Morken, Riyas Vettukattil, Ann-Mari Brubakk, Marius Widerøe

**Affiliations:** 1 Department of Laboratory Medicine, Children’s and Women’s Health, Norwegian University of Science and Technology, Trondheim, Norway; 2 Department of Pediatrics, St. Olavs University Hospital HF, Trondheim, Norway; 3 Department of Circulation and Medical Imaging, Norwegian University of Science and Technology, Trondheim, Norway; 4 Department of Ophthalmology, St. Olavs University Hospital HF, Trondheim, Norway; Hopital Robert Debre, FRANCE

## Abstract

Melatonin is a promising neuroprotective agent after perinatal hypoxic-ischemic (HI) brain injury. We used *in-vivo* ^1^H magnetic resonance spectroscopy to investigate effects of melatonin treatment on brain metabolism after HI. Postnatal day 7 Sprague-Dawley rats with unilateral HI brain injury were treated with either melatonin 10 mg/kg dissolved in phosphate-buffered saline (PBS) with 5% dimethyl sulfoxide (DMSO) or vehicle (5% DMSO and/or PBS) directly and at 6 hours after HI. ^1^H MR spectra from the thalamus in the ipsilateral and contralateral hemisphere were acquired 1 day after HI. Our results showed that injured animals had a distinct metabolic profile in the ipsilateral thalamus compared to sham with low concentrations of total creatine, choline, *N*-acetyl aspartate (NAA), and high concentrations of lipids. A majority of the melatonin-treated animals had a metabolic profile characterized by higher total creatine, choline, NAA and lower lipid levels than other HI animals. When comparing absolute concentrations, melatonin treatment resulted in higher glutamine levels and lower lipid concentrations compared to DMSO treatment as well as higher macromolecule levels compared to PBS treatment day 1 after HI. DMSO treated animals had lower concentrations of glucose, creatine, phosphocholine and macromolecules compared to sham animals. In conclusion, the neuroprotective effects of melatonin were reflected in a more favorable metabolic profile including reduced lipid levels that likely represents reduced cell injury. Neuroprotective effects may also be related to the influence of melatonin on glutamate/glutamine metabolism. The modulatory effects of the solvent DMSO on cerebral energy metabolism might have masked additional beneficial effects of melatonin.

## Introduction

Perinatal hypoxia-ischemia (HI) is a common cause of neonatal mortality and chronic neurodevelopmental disabilities, and is therefore a major health problem [1]. Reduced oxygen and glucose supply to the brain initiates an energy failure, release of excitatory amino acids into the synaptic cleft (glutamate excitotoxicity) and generation of reactive oxygen species leading to early cell death or necrosis. Re-oxygenation of the tissue after HI temporarily results in a restoration of energy reserves but also contributes to further oxidative stress to and from the mitochondria. The subsequent secondary energy failure after 6–48 hours is followed by a delayed injury cascade which is characteristic for HI in the neonatal brain with inflammation, apoptosis and delayed cell death [2].

The current treatment of infants with hypoxic-ischemic encephalopathy is supportive care and therapeutic hypothermia. A meta-analysis showed beneficial effects after therapeutic hypothermia in term or late preterm infants (≥ 35 gestational weeks) with moderate-to-severe HI injuries [3]. However, its efficacy is limited in the presence of inflammation [4] and it is not used in premature infants. Therefore, there is an urgent need for other treatments, of which melatonin is one of the most promising agents [5]. Melatonin is an endogenous neurohormone that freely crosses the placenta and blood-brain barrier, and it has been shown to be neuroprotective after neonatal HI injury in preclinical and clinical studies [5, 6]. The protective mechanisms of melatonin are multiple and putatively related to its anti-oxidative, anti-excitotoxic and anti-inflammatory properties. Melatonin and its metabolites are both direct free radical scavengers as well as indirect anti-oxidants by modulating pro- and anti-oxidative enzyme activity [7]. It has several protective effects on mitochondria, leading to increased ATP production and reduced oxidative stress [8]. Furthermore, the receptor-dependent anti-excitotoxic effects of melatonin limit the excessive calcium influx and, thereby, subsequent production of free radicals [9]. Further melatoninergic neuroprotection is achieved by the suppression of inflammatory processes after HI, for example the inhibition of NFκB activation [10] or the reduction of microglial density [11].

^1^H magnetic resonance spectroscopy (MRS) non-invasively provides information about several pathophysiological processes involved in HI simultaneously, and has proven to be a valuable prognostic biomarker in clinical neuroprotection trials [12]. Especially metabolic changes after HI in the deep gray matter are the most definite because of the high metabolic rate and the increased vulnerability for HI injury in these brain regions [1]. To date, the effect of melatonin treatment shortly after HI on metabolite concentrations obtained from ^1^H MRS in the deep gray matter is unknown.

The purpose of this study was to characterize the effects of early melatonin treatment on cerebral metabolism during the secondary energy failure. Based on the known effects of melatonin, we hypothesized that melatonin treatment after HI induces early beneficial metabolic changes related to energy metabolism, excitotoxic neurotransmission and anti-oxidant levels. With the use of absolute concentrations and multivariate analyses we show the potential in analyzing whole metabolic profiles as well as single metabolites. This provides information about the underlying metabolic processes and enables assessment of early treatment effects.

## Materials and methods

### Materials

#### Animals and drugs

Sprague-Dawley rats were bred in the Comparative Medicine Core Facility at the Norwegian University of Science and Technology in Trondheim. Dams and their pups were kept on a 12:12 hour light-dark cycle with food and water ad libitum. All animal experiments were performed according to European Union and Norwegian regulations and guidelines for animal experimentation and approved by the Norwegian authority on animal welfare (permit number: 4975).

Melatonin and dimethyl sulfoxide (DMSO) were purchased from Sigma-Aldrich (St. Louis, MA / Irvine, UK), isoflurane from Baxter Medication Delivery (Oslo, Norway), bupivacain from AstraZenica (Oslo, Norway) and phosphate-buffered saline (PBS) from Fisher Scientific (Oslo, Norway).

### Methods

#### Hypoxia-ischemia and experimental groups

The Vannucci model [13] for neonatal HI was used. In short, seven-day-old (P7) rat pups of both sexes (mean weight 15.9 g, SD 1.9 g) were anesthetized with isoflurane, their right carotid artery was identified and thermo-cauterized and, after a 2 hour recovery period, they were exposed to hypoxia (8% oxygen) for 105 minutes. Sham-operated littermates underwent the same procedure except for right carotid artery cauterization and hypoxia. The pups were randomly assigned to either HI (n = 30) or sham (n = 18) and one of three different treatment conditions before surgery. Directly after HI, they received an intraperitoneal injection with either (1) a 4 mg/ml solution of melatonin (MEL, 10 mg/kg) dissolved in PBS with 5% DMSO (HI+MEL n = 11, sham+MEL n = 6), (2) PBS with 5% DMSO (HI+DMSO n = 11, sham+DMSO n = 6) or (3) only PBS (HI+PBS n = 8, sham+PBS n = 6). Melatonin is only slightly soluble in saline therefore the solvent DMSO was used. All animals received a second injection 6 hours after the hypoxia.

#### *In-vivo* ^1^H MRS: Acquisition

*In-vivo* ^1^H MRS was performed on the day (15–24 hours) after HI on a 7T magnet (Bruker BioSpec 70/20, Bruker BioSpin, Ettlingen, Germany) using a 86 mm volume resonator for radio frequency transmission and a phased array mouse head surface coil for reception. During scanning the animals were anesthetized with isoflurane (3% induction, 1.5% maintenance) in a mixture of oxygen and air. They were placed in a prone position on a water-heated bed. The heads of the rats were fixated using a nose mask and styrofoam. The respiration and oxygen saturation were monitored.

Before ^1^H MRS acquisition, a first- and second-order shim was performed using FASTMAP procedure (Bruker BioSpin). A single voxel of 2.5 x 2.5 x 2.5 mm^3^ was placed in the thalamus of the right (ipsilateral to the lesion) and left (contralateral to the lesion) hemisphere using a T_2_-weighted image as reference. A point-resolved spatially localized spectroscopy (PRESS) sequence with water suppression using variable pulse power and optimized relaxation delays (VAPOR) was acquired; echo time (TE) 15 ms, repetition time (TR) 2500 ms, bandwidth 50 kHz, 2048 complex points, 128 averages, spectral resolution 0.98 Hz/pts. The use of a short echo time allows quantification of lipid concentrations. However, a disadvantage was that the lactate peak could not be reliably identified. Since the water signal was chosen as an internal standard for metabolite quantification a scan without water suppression for each spectroscopy voxel was obtained with the same parameters as the scans with water suppression (4 averages). The T_2_ relaxation time of brain water in the voxel was measured in a sample of both sham and HI animals (TE = 15, 45, 75, 105, 135, 165, 195, 225 ms, TR 5000 ms, rapid acquisition with relaxation enhancement (RARE) factor 2). Based on the assumption that the voxel only contained grey matter, a mono-exponential T_2_ curve fitting of the water signal provided correction factors for the attenuation of the MR-visible water signal.

#### *In-vivo* ^1^H MRS: Analysis

Metabolite concentrations were quantified using LCModel [14]. Eddy current corrections were applied and the unsuppressed water signal was used as an internal reference for water scaling. The P7 rat brain has a water concentration of 87.7% which corresponds to 48.7M (48674 mM) [15]. It has been reported that the brain water content only increases with 2% 24 hours after severe HI brain injury caused by 3 hours of hypoxia [16]. Because of the shorter hypoxia period in our study versus that of Mujsce et al. (1990) and the variability in lesion size in the Vannucci model, we used the same water concentration in all animals. This may lead to an underestimation of the metabolite concentration up to 2% in the severely injured animals. Due to differences in the T_2_ relaxation of water, different correction factors (ATTH_2_O) were applied based on the extent of injury as assessed on T_2_-weighted images. Finally, the concentrations were exported to Microsoft Excel and corrected for the number of scans and the total receiver gain, as this is not done automatically for Bruker data in LCModel (correction factor 0.03125).

**Fig 1:**
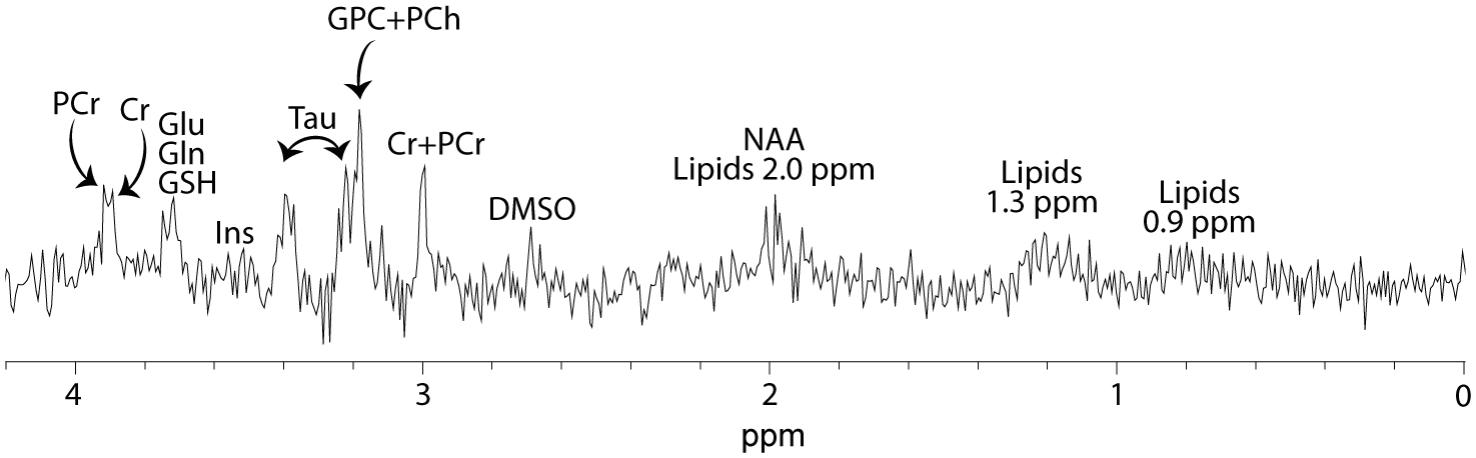
^1^H spectrum of the ipsilateral thalamus. Acquired ^1^H spectrum of the ipsilateral thalamus of an animal exposed to HI. Cr, creatine; DMSO, dimethyl sulfoxide; Gln, glutamine; Glu, glutamate; GPC, glycerophosphocholine; GSH, glutathione; Ins, myo-inositol; NAA, N-acetyl aspartate; PCh, phosphocoline; PCr, phosphocreatine; Tau, taurine.

Six spectra with a signal-to-noise ratio (SNR) < 3 or/and a linewidth of > 0.05 ppm were excluded (ipsilateral thalamus of 1 Sham+PBS, 2 Sham+MEL, 1 HI+MEL and bilaterally of 1 HI+PBS). One pup was in a poor clinical condition and scanning was terminated before the ^1^H MRS acquisition (HI+PBS). One spectrum of the contralateral thalamus is missing due to technical problems (HI+DMSO). In the end, we included 42 spectra of the ipsilateral thalamus (5 Sham+PBS, 6 Sham+DMSO, 4 Sham+MEL, 6 HI+PBS, 11 HI+DMSO, 10 HI+MEL) and 45 spectra of the contralateral thalamus (6 Sham+PBS, 6 Sham+DMSO, 6 Sham+MEL, 6 HI+PBS, 10 HI+DMSO, 11 HI+MEL) in the final analyses. The spectra had an overall SNR of 7 and a linewidth of 0.026 ppm. An example of a ^1^H MR spectrum of the ipsilateral thalamus is shown in Fig 1.

#### Statistical analysis

We used Principal Component Analysis (PCA) to explore and visualize how a set of metabolites from one subject varied from others [17]. In the score plot of principle component (PC) 1 and 2, which describe most of the variation, samples with a similar metabolic profile will cluster, while the corresponding loading profile displays the importance of each variable within the PC. PCA was performed in R (R Core Team, 2016, R: A language and environment for statistical computing. R Foundation for Statistical Computing, Vienna, Austria). Univariate statistical analyses were performed in SPSS (version 22.0; IBM, Chicago, IL). The metabolite concentrations were not normally distributed. A Kruskal Wallis test with Mann-Whitney U tests for pairwise comparisons was therefore used to test for differences in metabolite concentrations between the different groups within each hemisphere. All p-values were corrected by the false discovery rate control method (Benjamini & Hochberg's method) with *p* < 0.05 as a reference level of significance. The magnitude of the metabolite changes between the treatment groups in the sham animals were much smaller than those compared to HI. Therefore, the sham animals were considered as one group when the HI animals were compared to the sham animals.

## Results

### Different metabolic profiles of the ipsilateral thalamus in HI and sham-operated animals

**Fig 2:**
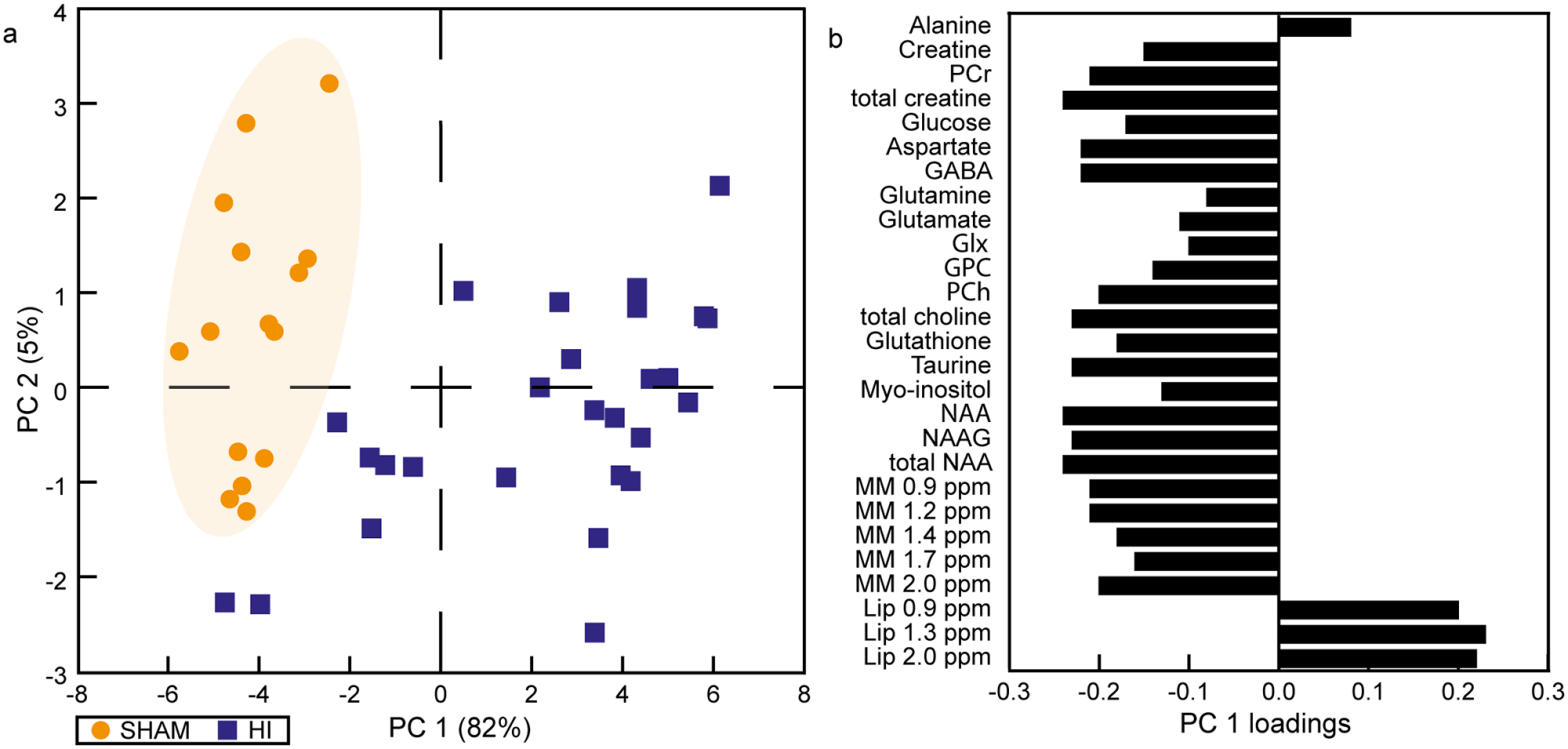
Metabolic profiles of the ipsilateral thalamus of HI and sham animals 1 day after HI. (a) The PCA score plot shows the metabolic profiles of all animals. The HI animals were separated from the sham animals with the corresponding loading plot of PC 1 (b) showing the metabolic alterations that caused this separation. The shaded area is drawn based on visual inspection and is meant to highlight the separation between the groups. Glx, glutamine+glutamate; GPC glycerophosphocholine; HI, hypoxia-ischemia; NAA, N-acetyl aspartate; NAAG, N-acetyl aspartyl glutamate; MM, macromolecules; Lip, lipids; PC, principle component; PCA, principle component analysis; PCh, phosphocholine; PCr, phosphocreatine; ppm, parts per million.

**Fig 3:**
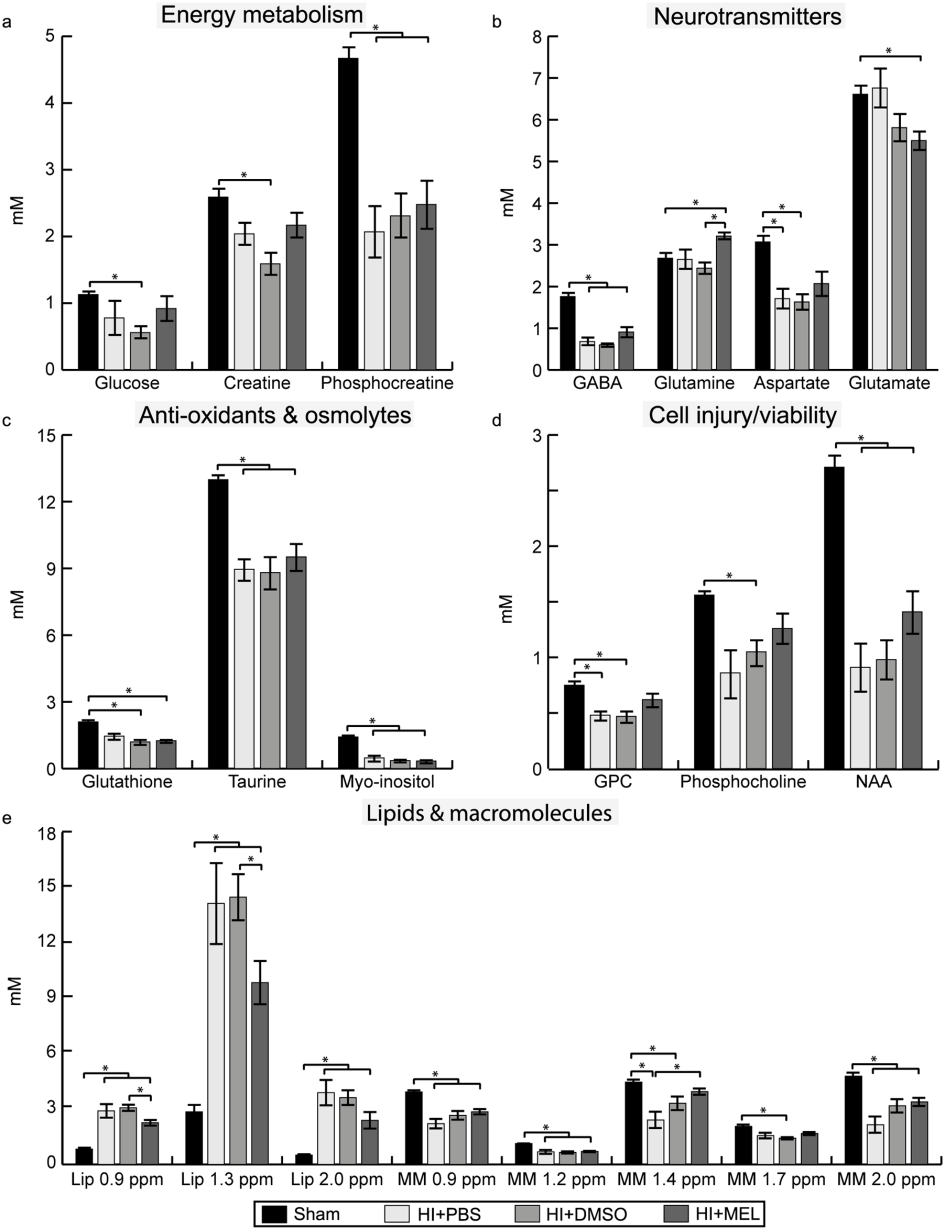
Metabolite concentrations in the ipsilateral thalamus within 1 day after HI. Concentrations of (a) energy metabolites, (b) neurotransmitters, (c) anti-oxidants and osmolytes, (d) metabolites representing cell injury/viability and (e) lipids and macromolecules in HI animals exposed to PBS, DMSO or melatonin (MEL) treatment and sham animals (here presented as one group). Results are presented as mean ± SEM. * significant differences between groups. GPC glycerophosphocholine; HI, hypoxia-ischemia; Lip, lipids; MM, macromolecules; NAA, N-acetyl aspartate; ppm, parts per million; sham, sham-operated.

The PCA showed a clear separation between the metabolic profile of HI and sham animals (Fig 2a). The corresponding loading plot of PC 1, which described 82% of the variation, showed that the distinction between HI and sham is mainly due to higher levels of lipids and lower concentrations of NAA, total choline, and total creatine (Cr) in the HI animals (Fig 2b). When comparing absolute concentrations, HI animals had lower concentrations of phosphocreatine (PCr) and total creatine versus sham animals (Fig 3, S1 Table). In addition, the HI animals had reduced concentrations of the osmolytes taurine and myo-inositol as well as lower levels of the neurotransmitter GABA compared to sham animals. However, the levels of the neurotransmitters glutamate and glutamine in HI animals treated with DMSO or PBS were similar to sham. The levels of NAA, total choline and macromolecules, metabolites that are thought to reflect cell integrity, were lower in HI animals versus sham animals while lipids were higher (Fig 3, S1 Table).

The ^1^H spectra were obtained between 15 and 24 hours after the end of hypoxia. There were no significant correlations between metabolite concentrations and time after HI except for the combined glutamine+glutamate concentration in HI+DMSO/PBS animals, which was negatively correlated with time after HI (*r* -0.587, *p* 0.013).

### Metabolic changes after melatonin treatment in the ipsilateral thalamus

**Fig 4:**
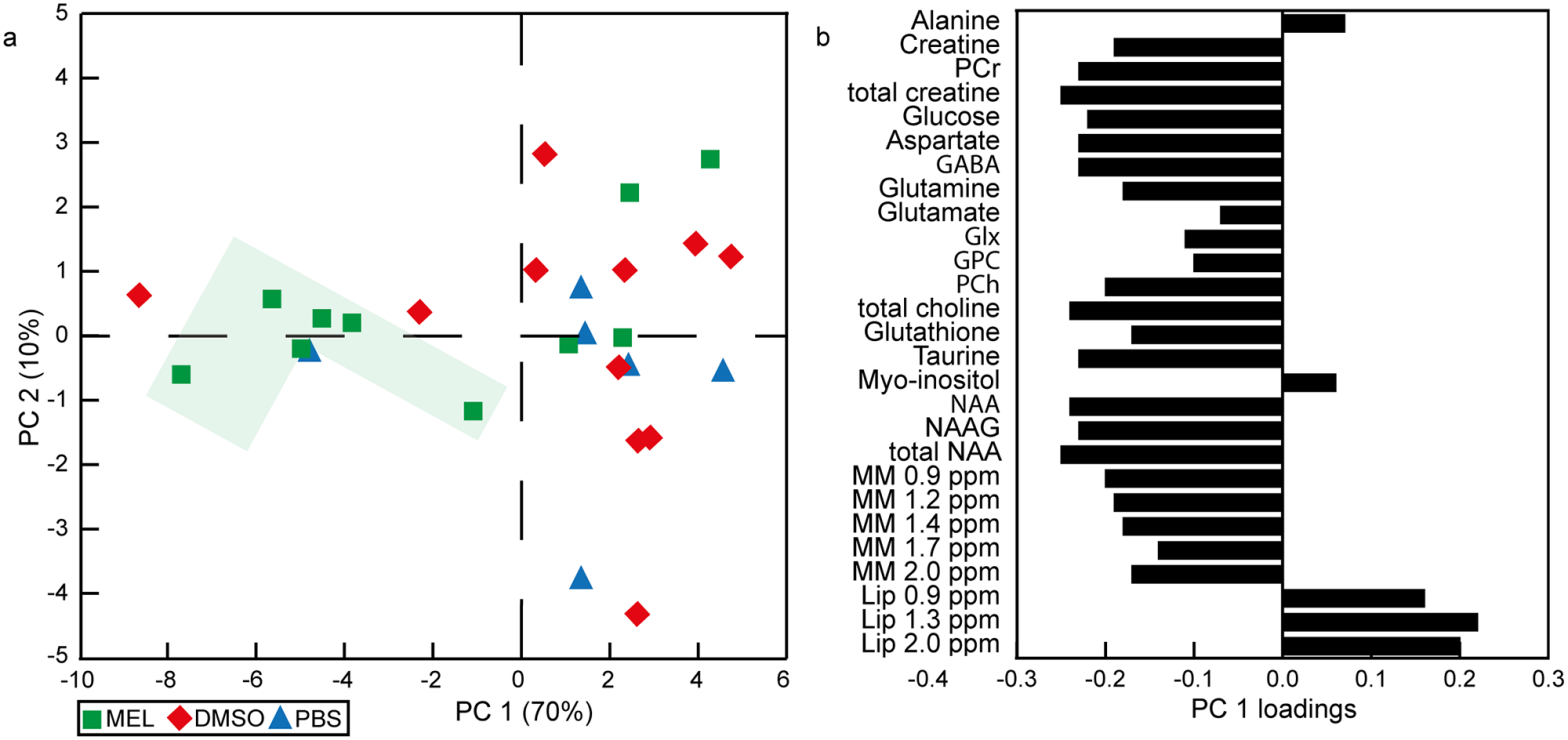
Metabolic profiles of the ipsilateral thalamus of HI animals after 2 treatment injections. (a) The metabolic profiles of the HI animals treated with either melatonin (10 mg/kg) dissolved in PBS with 5% DMSO (MEL), PBS with 5% DMSO (DMSO) or only PBS (PBS) are displayed in the PCA score plot. The shaded area marks the animals treated with melatonin with higher levels of energy metabolites, neurotransmitters and macromolecules as shown in (b). (b) The corresponding loading plot shows the metabolic alterations related to the separation along PC 1. Glx, glutamine+glutamate; GPC glycerophosphocholine; HI, hypoxia-ischemia; NAA, N-acetyl aspartate; NAAG, N-acetyl aspartyl glutamate; MM, macromolecules; Lip, lipids; PC, principle component; PCA, principle component analysis; PCh, phosphocholine; PCr, phosphocreatine; ppm, parts per million.

The PCA showed a separation of HI animals with mild and severe injury along the PC 1 where a more favorable metabolic profile was reflected in higher levels of total creatine, choline and NAA, and lower levels of lipids (Fig 4). More HI animals treated with melatonin had a favorable metabolic profile (6 out of 10) compared to DMSO (2 out of 11) and PBS (1 out of 6). The HI animals treated with melatonin had a higher absolute concentration of glutamine and a lower amount of lipids at 0.9 and 1.3 ppm than HI+DMSO animals. Furthermore, HI+MEL animals had a higher concentration of macromolecules at 1.4 ppm compared to HI+PBS animals as well as lower glutamate levels compared to sham animals. HI+MEL animals had also a lower ratio of glutamate over glutamine (mean 1.7, SEM 0.1) compared to HI+DMSO (mean 2.4, SEM 0.2 *p* < 0.001), HI+PBS (mean 2.5, SEM 0.3 *p* 0.027) and sham animals (mean 2.5, SEM 0.1, *p* < 0.001). In addition, HI+DMSO and HI+PBS animals had reduced levels of aspartate and glycerophosphocholine (GPC) whereas these concentrations were similar to sham in HI+MEL animals (Fig 3).

Although there were no major alterations in the metabolic profiles of sham animals after treatment, the ones treated with melatonin had lower glutathione concentrations (mean 1.48, SEM 0.13) compared to sham animals treated with DMSO (mean 2.17, SEM 0.25, *p* 0.033) or PBS (mean 2.51, SEM 0.10, *p* 0.014).

### Metabolic effects of DMSO in the ipsilateral thalamus

DMSO was detectable at 2.74 ppm in the ^1^H MR spectra of all animals treated with DMSO or melatonin. HI animals treated with DMSO had lower concentrations of glucose, creatine, phosphocholine and macromolecules at 1.7 ppm compared to sham animals whereas these concentrations were not different from sham in HI+MEL or HI+PBS animals (Fig 3, S2 Table). In addition, HI+DMSO and HI+MEL animals had lower glutathione levels compared to sham. Sham animals given DMSO (mean 1.54, SEM 0.10, *p* 0.011) or melatonin (mean 1.50, SEM 0.07, *p* 0.014) had lower levels of GABA than those treated with PBS (mean 2.23, SEM 0.20).

### Metabolite changes in the contralateral thalamus

**Fig 5:**
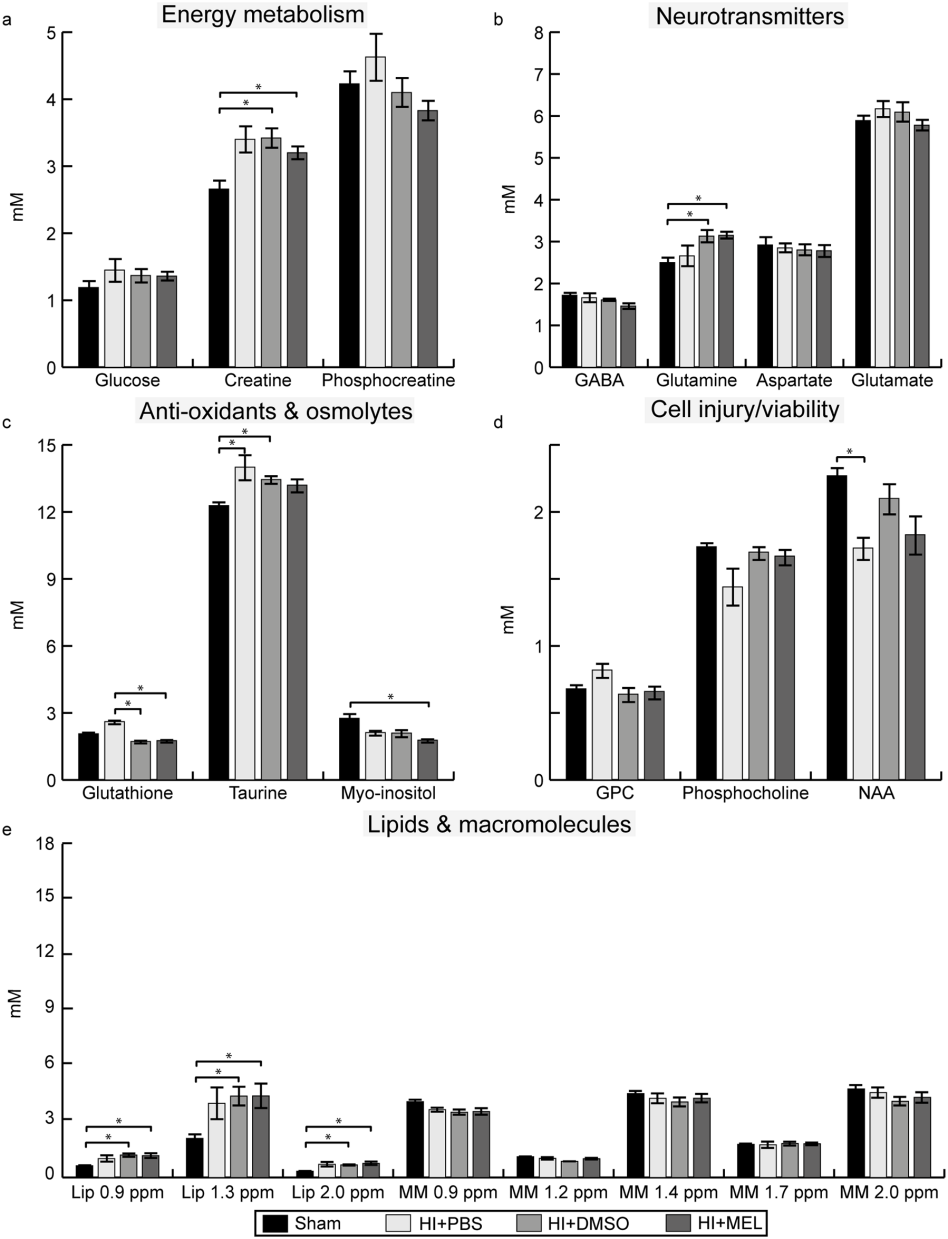
Metabolite concentrations in the contralateral thalamus within 1 day after HI. Concentrations of (a) energy metabolites, (b) neurotransmitters, (c) anti-oxidants and osmolytes, (d) metabolites representing cell injury/viability and (e) lipids and macromolecules in HI animals exposed to PBS, DMSO or melatonin (MEL) treatment and sham animals (here presented as one group). Results are presented as mean ± SEM. * significant differences between groups. GPC glycerophosphocholine; HI, hypoxia-ischemia; Lip, lipids; MM, macromolecules; NAA, N-acetyl aspartate; ppm, parts per million; sham, sham-operated.

The metabolic changes in the contralateral thalamus were of a smaller magnitude compared to the ipsilateral hemisphere and there was not a clear separation between the metabolic profiles of the HI and sham groups (Fig 5). However, an effect of HI was visible as higher concentrations of creatine and lipids at 0.9, 1.3 and 2.0 ppm in the HI+DMSO and HI+MEL animals compared to sham, with similar trends seen in HI+PBS. In addition, the NAA concentration was lower in HI+PBS than in sham animals. HI+DMSO and HI+MEL animals had higher glutamine concentrations compared to sham as well as lower concentrations of glutathione compared to HI+PBS animals. Furthermore, the concentration of taurine was higher in HI+DMSO and HI+PBS animals compared to sham animals whereas the concentration of myo-inositol was lower in HI+MEL animals compared to sham animals. There were no differences between HI+MEL and HI+DMSO animals.

Like in the ipsilateral thalamus, the sham animals treated with melatonin had lower glutathione concentrations (mean 1.75, SEM 0.22) compared to sham animals treated with PBS (mean 2.48, SEM 0.13, *p* 0.025). Sham animals treated with DMSO (mean 1.57, SEM 0.08, *p* 0.025) or melatonin (mean 1.52, SEM 0.04, *p* 0.004) had lower levels of GABA than those treated with PBS (mean 2.07, SEM 0.13). Moreover, the sham animals treated with DMSO had lower levels of PCr (mean 4.74, SEM 0.23, *p* 0.018) compared to the ones treated with PBS (mean 3.63, SEM 0.23).

## Discussion

### Altered metabolism in the thalamus after neonatal hypoxia-ischemia

The use of metabolomics enables studies of key processes of cerebral metabolism that are affected during the evolving injury cascade after neonatal HI, including energy metabolism, neurotransmission, and osmolyte and anti-oxidant levels. The reduction of free choline and phosphocholine in the ipsilateral thalamus of HI animals compared to sham as found in this study points towards a decreased cellularity and/or apoptosis [18] since these metabolites are phospholipid metabolism intermediates and are involved in the synthesis and breakdown of cell membranes [19]. The finding of reduced PCr and total creatine in the ipsilateral thalamus following HI probably reflects the continuing loss or degradation of creatine as a consequence of brain cell injury [20]. However, a compensatory increase of (total) creatine was seen in the HI animals in the contralateral thalamus. In addition, the HI animals had lower levels of NAA and NAAG, metabolites that are mainly present in neurons but also in oligodendroglia progenitors [21]. A reduction of these metabolites after HI has also been reported by others [22, 23] and has been associated with reduced neuronal viability, function and density [19].

The metabolite changes related to cell damage and impaired energy metabolism found in the present study were accompanied by metabolic alterations presumably representing different pathological processes contributing to final brain injury. Decreased concentrations of glutathione and taurine may indicate a reduced anti-oxidant defense in the injured neonatal brains 1 day after HI [24, 25]. Taurine also functions as an osmolyte. Thus, the decreased concentrations of taurine and myo-inositol in the ipsilateral thalamus found in the present study may reflect an impaired water homeostasis in response to osmotic stress after HI. We did not find changes in glutamine and glutamate levels in the ipsilateral thalamus of HI+PBS/DMSO compared to sham animals. This corresponds well with other studies showing normalization of glutamine and glutamate levels during the first hours after HI [26, 27]. Moreover, the concentration of the neurotransmitter GABA was decreased in the ipsilateral thalamus of the HI animals in the present study. This is in agreement with findings by others [23, 28] and might suggest a specific vulnerability of GABAergic neurons to HI.

### Changes in lipid and macromolecule concentrations after HI

The advantage of using a short echo time is the possibility to reliably quantify lipid concentrations. Important findings in this study were the increased lipid levels in the ipsilateral and contralateral thalamus and the decreased macromolecule levels in the ipsilateral thalamus after HI. Although the precise underlying substrates are not well known, an increased amount of lipids is, like choline, considered a sign of membrane degradation with release of fatty acids [29]. The largest increase in lipid peaks was observed at 1.3 ppm which arise from methylene groups (-CH_2_) in mobile fatty acids. In the parietotemporal area, this peak has been positively correlated with apoptotic and necrotic cell death and a loss of mitochondrial membrane potential at 1 day after HI in neonatal rats [30]. Furthermore, it is suggested that the macromolecule resonances represent cytosolic proteins that can be liberated after cell injury. In perinatal hypoxia-ischemia, macromolecular synthesis has been described as a differentiating factor between necrosis and apoptosis with a decreased synthesis in necrosis and an increased synthesis in apoptosis [31]. The reduced levels of macromolecules 1 day after HI might be explained by a reduced synthesis of macromolecules in the early hours after HI due to necrosis combined with an elevated degradation of liberated cytosolic proteins because of cell injury.

### Treatment effects: Melatonin

Within a day after HI, the majority of the HI animals treated with 2 injections of melatonin had a metabolic profile in the ipsilateral thalamus that was more similar to sham and characterized by higher levels of total creatine, choline and NAA, and lower levels of lipids than most of the vehicle-treated HI animals. When we compared absolute metabolite concentrations, melatonin treated animals showed signs of less cell injury compared to DMSO and PBS treated animals reflected in lower concentration of lipids and higher concentration of macromolecules in the ipsilateral thalamus. These findings are in agreement with other preclinical studies that have shown neuroprotection with melatonin treatment after HI [6]. However, it contrasts our previous findings at 1 hour after HI where no effects of melatonin on neuronal or glial mitochondrial metabolism were observed [32]. Different brain regions, a longer interval between melatonin treatment after HI and the time-point of evaluation, as well as 2 treatment injections instead of 1 are potential factors that may explain the different outcome in our previous report. Based on this, it is conceivable that either the therapeutic window of melatonin treatment is longer than 1 hour or that multiple doses are necessary for neuroprotection.

The mechanisms behind melatoninergic neuroprotection are not completely clear. Our data suggest that melatonin plays a role in the regulation of (excitatory) neurotransmitter levels as shown by the increased glutamine levels among HI+MEL animals and the lower glutamate-glutamine ratio compared to HI+PBS, HI+DMSO and sham animals in the ipsilateral thalamus. Although we do not have information about cellular compartmentalization, the increased glutamine in the thalamus of HI+MEL animals possibly indicate that the ATP-dependent synthesis of glutamine from glutamate in astrocytes is highly active. This might contribute to the neuroprotective effects of melatonin through the normalization of excitatory neurotransmitter levels in the synapse and/or the supply of glutamine that can be used as an alternative energy substrate for mitochondria [33]. The lower amount of glutamate in HI+MEL compared to sham animals in the ipsilateral thalamus might be beneficial by decreasing seizure activity or limiting cerebral energy consumption for neurotransmission. Whether this modulation of neurotransmitter concentrations is the direct result of melatonin acting on synaptic proteins or caused by an upstream mechanism remains an interesting question for further study. Further, it is not possible to exclude an effect of the solvent DMSO, since the glutamate concentrations in HI+DMSO and HI+MEL are similar and we previously reported a reduced transfer of glutamine from astrocytes to neurons leading to less glutamate in HI+DMSO animals [32].

### Treatment effects: DMSO

The presence of DMSO in the ^1^H MR spectra and the metabolic changes observed in the animals treated with DMSO suggest that DMSO affects cerebral metabolism. It is still a topic of debate whether DMSO is neurotoxic or neuroprotective for the brain [34, 35]. In this study, DMSO treatment resulted in reduced PCr and GABA concentrations in sham animals as well as decreased levels of glucose and creatine in HI animals. This may reflect toxic effects of DMSO on energy metabolism and neurotransmitter synthesis, possibly by its detrimental effects on mitochondrial integrity and function [32, 36]. The reduced levels of glutathione in HI animals after DMSO treatment might either be attributed to its perceived anti-oxidative properties [34] and, consequently, a reduced need for glutathione production, or to a diminished anti-oxidant defense with DMSO treatment. Our findings stress the importance of including appropriate controls groups when performing metabolic studies.

### Strengths and limitations

A strength of the present study is the use of single metabolite concentrations to characterize the effects of melatonin treatment after neonatal HI. Previous studies have mostly utilized ratios such as NAA/lactate or NAA/Cr as prognostic MRS biomarkers for treatment response without absolute quantification of individual metabolites [37, 38]. This approach is susceptible to misinterpretation because the denominator (often creatine) is altered following HI as found in the present study and also reported by others [18, 39]. However, our study has several possible limitations. First, the ^1^H spectra were obtained from the thalamus, an area that is known to be severely injured in this animal model, which made it challenging to acquire spectra from the ipsilateral thalamus with a good signal-to-noise ratio. Despite this, the model spectra in LCModel were able to quantify the metabolite peaks with an estimated standard deviation of ≤ 50%SD in most of the metabolites and ≤ 25%SD in the central metabolites (e.g. creatine, choline, glutamine, glutamate, glutathione, taurine). Second, the ^1^H spectra were acquired between 15 and 24 hours after HI. During this period, rapid metabolic changes related to a secondary energy failure occur and this might have influenced the results. However, except for the correlation between the glutamate+glutamine concentration and the time after HI, we did not find any correlation for the other metabolites. Finally, the poor solubility of melatonin required the use of the solvent DMSO. Unfortunately, there is evidence that DMSO itself modulates cerebral metabolism [40]. To account for this, HI and sham control groups treated with DMSO were included in the study. More work is needed to develop parenteral melatonin formulations without biologically active solvents, to simplify interpretation of results and help transition into clinical studies.

### Conclusion and clinical implications

^1^H MRS in the thalamus within 1 day after HI identified distinct metabolic profiles in injured versus non-injured animals. The reduced levels of creatine, NAA and choline, and the increased levels of lipids in the injured animals might reflect impaired energy metabolism, membrane degeneration and neuronal cell death. Animals treated with melatonin showed low lipid concentrations indicating reduced cell death as well as a potential beneficial increase in glutamine. However, the effects of melatonin might be partly influenced by modulatory effects of the solvent DMSO. The need for the identification of a nontoxic solvent for melatonin is essential before melatonin can be utilized in clinical practice. Further studies should optimize the melatonin treatment regimen by evaluating the therapeutic plasma concentration leading to significant neuroprotection as well as the best administration route. Based on the present study, a dosage higher than 10 mg/kg with multiple administrations during the first 24 hours, and probably a prolonged duration of treatment are needed to achieve maximum benefit of melatonin treatment.

## Supporting information

S1 Table Metabolite concentrations in the ipsilateral thalamus. Metabolite concentrations in the ipsilateral thalamus after 2 treatment injections. Sham and HI animals were treated with either melatonin dissolved in PBS with 5% DMSO (HI+MEL, n = 10), PBS with 5% DMSO (HI+DMSO, n = 11) or only PBS (HI+PBS, n = 6) immediately and 6 hours after HI. The sham animals (sham+MEL, sham+DMSO, sham+PBS, n = 15) are considered as one group because the magnitude of the metabolite changes between the treatment groups in the sham animals were much smaller compared to those in HI animals. Differences between the groups were evaluated using a Kruskal Wallis test with Mann-Whitney U tests for pairwise comparisons. *P*-values were corrected for multiple comparisons by the false discovery rate control method. *Significant differences between HI and sham animals. ^§^ Significant difference between HI+MEL and HI+DMSO animals. ‡ Significant difference between HI+MEL and HI+PBS animals. HI, hypoxia-ischemia; sham, sham-operated; Glx, glutamate+glutamine; GPC, glycerophosphocholine; NAA, N-acetyl aspartate; NAAG, N-acetyl aspartyl glutamate; MM, macromolecules; Lip, lipids; ppm, parts per million.
(DOCX)

S2 Table Metabolite concentrations in the contralateral thalamus. Metabolite concentrations in the contralateral thalamus after 2 treatment injections. Sham and HI animals were treated with either melatonin dissolved in PBS with 5% DMSO (HI+MEL, n = 10), PBS with 5% DMSO (HI+DMSO, n = 11) or only PBS (HI+PBS, n = 6) immediately and 6 hours after HI. The sham animals (sham+MEL, sham+DMSO, sham+PBS, n = 15) are considered as one group because the magnitude of the metabolite changes between the treatment groups in the sham animals were much smaller compared to those in HI animals. Differences between the groups were evaluated using a Kruskal Wallis test with Mann-Whitney U tests for pairwise comparisons. *P*-values were corrected for multiple comparisons by the false discovery rate control method. * Significant differences between HI and sham animals. ^§^ Significant difference between HI+MEL and HI+DMSO animals. ‡ Significant difference between HI+MEL and HI+PBS animals. ¶ Significant difference between HI+PBS and HI+DMSO animals. HI, hypoxia-ischemia; sham, sham-operated; Glx, glutamate+glutamine; GPC, glycerophosphocholine; NAA, N-acetyl aspartate; NAAG, N-acetyl aspartyl glutamate; MM, macromolecules; Lip, lipids; ppm, parts per million.
(DOCX)
