# Supplementary material for: Early metabolite changes after melatonin treatment in neonatal rats with hypoxic-ischemic brain injury studied by in-vivo 1H MR spectroscopy
Source: PLoS One. 2017 Sep 21;12(9):e0185202. doi: 10.1371/journal.pone.0185202 (PMC5608359; doi:10.1371/journal.pone.0185202)
Supplement: S1 Table — Metabolite concentrations in the ipsilateral thalamus after 2 treatment injections. Sham and HI animals were treated with either melatonin dissolved in PBS with 5% DMSO (HI+MEL, n = 10), PBS with 5% DMSO (HI+DMSO, n = 11) or only PBS (HI+PBS, n = 6) immediately and 6 hours after HI. The sham animals (sham+MEL, sham+DMSO, sham+PBS, n = 15) are considered as one group because the magnitude of the metabolite changes between the treatment groups in the sham animals were much smaller compared to those in HI animals. Differences between the groups were evaluated using a Kruskal Wallis test with Mann-Whitney U tests for pairwise comparisons. P-values were corrected for multiple comparisons by the false discovery rate control method. *Significant differences between HI and sham animals. § Significant difference between HI+MEL and HI+DMSO animals. ‡ Significant difference between HI+MEL and HI+PBS animals. HI, hypoxia-ischemia; sham, sham-operated; Glx, glutamate+glutamine; GPC, glycerophosphocholine; NAA, N-acetyl aspartate; NAAG, N-acetyl aspartyl glutamate; MM, macromolecules; Lip, lipids; ppm, parts per million. (DOCX) [file pone.0185202.s001.docx]

**Table S1 Metabolite concentrations in the ipsilateral thalamus (mM)**

|  | SHAM | |  | HI+PBS | |  | HI+DMSO | |  | HI+MEL | |
| --- | --- | --- | --- | --- | --- | --- | --- | --- | --- | --- | --- |
|  | Mean | SEM |  | Mean | SEM |  | Mean | SEM |  | Mean | SEM |
| **Energy metabolism** |  |  |  |  |  |  |  |  |  |  |  |
| Alanine | 1.06 | 0.10 |  | **1.66*** | 0.14 |  | 1.35 | 0.14 |  | 1.26 | 0.09 |
| Glucose | 1.13 | 0.06 |  | 0.78 | 0.27 |  | **0.56*** | 0.10 |  | 0.92 | 0.20 |
| Creatine | 2.59 | 0.14 |  | 2.04 | 0.18 |  | **1.59*** | 0.18 |  | 2.17 | 0.20 |
| Phosphocreatine | 4.67 | 0.18 |  | **2.07*** | 0.40 |  | **2.31*** | 0.34 |  | **2.48*** | 0.37 |
| Total creatine | 7.31 | 0.15 |  | **4.12*** | 0.47 |  | **3.93*** | 0.44 |  | **4.65*** | 0.53 |
| **Neurotransmitters** |  |  |  |  |  |  |  |  |  |  |  |
| GABA | 1.76 | 0.12 |  | **0.68*** | 0.11 |  | **0.60*** | 0.06 |  | **0.91*** | 0.14 |
| Aspartate | 3.07 | 0.18 |  | **1.71*** | 0.26 |  | **1.63*** | 0.21 |  | 2.07 | 0.31 |
| Glutamine | 2.68 | 0.14 |  | 2.65 | 0.25 |  | 2.44 | 0.16 |  | **3.21*^§^** | 0.10 |
| Glutamate | 6.61 | 0.22 |  | 6.76 | 0.49 |  | 5.81 | 0.35 |  | **5.50*** | 0.25 |
| Glx | 9.30 | 0.31 |  | 9.89 | 0.57 |  | 8.26 | 0.44 |  | 8.71 | 0.34 |
| **Anti-oxidants & osmolytes** | |  |  |  |  |  |  |  |  |  |  |
| Glutathione | 2.10 | 0.15 |  | 1.46 | 0.18 |  | **1.20*** | 0.15 |  | **1.25*** | 0.11 |
| Taurine | 12.99 | 0.27 |  | **8.96*** | 0.52 |  | **8.81*** | 0.77 |  | **9.52*** | 0.65 |
| Myo-inositol | 1.42 | 0.14 |  | **0.48*** | 0.17 |  | **0.36*** | 0.11 |  | **0.34*** | 0.12 |
| **Cell injury/viability** |  |  |  |  |  |  |  |  |  |  |  |
| GPC | 0.75 | 0.05 |  | **0.48*** | 0.05 |  | **0.47*** | 0.06 |  | 0.62 | 0.07 |
| Phosphocholine | 1.56 | 0.05 |  | 0.86 | 0.23 |  | **1.05*** | 0.13 |  | 1.26 | 0.15 |
| Total choline | 2.31 | 0.05 |  | **1.39*** | 0.22 |  | **1.47*** | 0.15 |  | **1.76*** | 0.17 |
| NAA | 2.71 | 0.12 |  | **0.91*** | 0.22 |  | **0.98*** | 0.19 |  | **1.41*** | 0.20 |
| NAAG | 1.04 | 0.05 |  | **0.16*** | 0.10 |  | **0.25*** | 0.08 |  | **0.55*** | 0.14 |
| Total NAA | 3.75 | 0.12 |  | **1.07*** | 0.28 |  | **1.23*** | 0.26 |  | **1.96*** | 0.33 |
| **Lipids & macromolecules** | |  |  |  |  |  |  |  |  |  |  |
| Lip 0.9 ppm | 0.83 | 0.13 |  | **2.90*** | 0.42 |  | **3.07*** | 0.22 |  | **2.26*^§^** | 0.21 |
| Lip 1.3 ppm | 2.85 | 0.44 |  | **14.14*** | 2.25 |  | **14.48*** | 1.30 |  | **9.85*^§^** | 1.23 |
| Lip 2.0 ppm | 0.51 | 0.10 |  | **3.89*** | 0.74 |  | **3.62*** | 0.45 |  | **2.39*** | 0.50 |
| MM 0.9 ppm | 3.93 | 0.14 |  | **2.21*** | 0.31 |  | **2.66*** | 0.29 |  | **2.85*** | 0.19 |
| MM 1.2 ppm | 1.13 | 0.06 |  | **0.68*** | 0.16 |  | **0.65*** | 0.11 |  | **0.70*** | 0.09 |
| MM 1.4 ppm | 4.46 | 0.19 |  | **2.41*** | 0.50 |  | **3.31*** | 0.42 |  | **3.95^ǂ^** | 0.23 |
| MM 1.7 ppm | 2.06 | 0.14 |  | 1.56 | 0.20 |  | **1.42*** | 0.11 |  | 1.68 | 0.13 |
| MM 2.0 ppm | 4.78 | 0.25 |  | **2.15*** | 0.49 |  | **3.18*** | 0.42 |  | **3.39*** | 0.28 |
